# Childhood trauma and cognitive decline in women: an empirical analysis of cognitive vulnerability in later life

**DOI:** 10.3389/fnagi.2026.1848025

**Published:** 2026-09-28

**Authors:** Mai A. Salem, Eid Abo Hamza, Dalia Bedewy, Reham A Amer, Abdalla A. R. M. Hamid, Emad Mahgoub, Ahmed A Moustafa

**Affiliations:** 1 Department of Neurology and Psychiatry, Faculty of Medicine, Tanta University, Tanta, Egypt; 2 College of Arts, Humanities and Social Sciences, University of Sharjah, Sharjah, United Arab Emirates; 3 Department of Mental Health, Faculty of Education, Tanta University, Tanta, Egypt; 4 Department of Psychology, College of Humanities and Sciences, Ajman University, Ajman, United Arab Emirates; 5 Humanities and Social Sciences Research Center (HSSRC), Ajman University, Ajman, United Arab Emirates; 6 Department of Educational Psychology, Faculty of Education, Tanta University, Tanta, Egypt; 7 Department of Clinical Psychology, College of Medicine & Health Sciences, United Arab Emirates University, Al Ain, United Arab Emirates; 8 Department of Clinical Psychology, Imam Abdulrahman Bin Faisal University, Dammam, Saudi Arabia; 9 Department of Human Anatomy and Physiology, Faculty of Health Sciences, University of Johannesburg, Johannesburg, South Africa; 10 Faculty of Psychology, University College of Professional Education, Wroclaw, Poland; 11 Centre for Data Analytics & School of Psychology, Bond University, Gold Coast, QLD, Australia

**Keywords:** adverse childhood experiences, Alzheimer’s disease, childhood trauma, cognitive health, MoCA, neglect, women

## Abstract

Childhood trauma has lasted consequences for women’s cognitive health and this may contribute to memory decline and increased vulnerability to cognitive impairment in later life. A cross sectional study was carried out to assess the correlation between childhood trauma and cognitive status among 55 women aged 58–79 years were selected from Departments of Psychiatry and General Medicine, Tanta University Hospital, Egypt. The Montreal Cognitive Assessment (MoCA) was used to evaluate cognitive performance, the Global Deterioration Scale (GDS) for global clinical staging, and the Childhood Trauma Questionnaire (CTQ) for childhood adversity. Overall, emotional abuse was exposed the most with a high mean score on the CTQ subscale. Pearson correlations and multiple linear regression models (controlling for age) showed that emotional neglect and physical neglect were the strongest predictors across both outcomes. For MoCA performance, emotional neglect (β = −0.0877, *r* = −0.85, *p* < 0.01) and physical neglect (β = −0.0100, *r* = −0.80, *p* < 0.01) showed the largest effects. GDS coefficients had an absolute value that was proportionately smaller (emotional neglect: β = 0.0112; physical neglect: β = 0.0013) and, consistent with the higher-is-worse nature of the GDS, were positive in direction. Additional findings show a greater amount of neglect predicted more advanced clinical staging, and that there was no change to the relative order of effects by predictor compared to the MoCA model. The regression model accounted for 72.5% of the variance (R^2^ = 0.725) in MoCA. The results indicate that childhood neglect, especially emotional neglect, might be an important risk factor for cognitive vulnerability in adulthood and highlight the necessity to intervene early in childhood to promote cognitive health in women.

## 1 Introduction

Alzheimer’s disease (AD) is a progressive neurodegenerative disorder that primarily affects older adults. This leads to declines in memory, cognitive abilities, and everyday functioning. It is the most common cause of dementia which has affected an estimated 50 million people worldwide. This figure expected to rise substantially as global populations age (Alzheimer’s Association, 2022; Scheidt, 2023). Epidemiological evidence has consistently demonstrated that women are more prone to developing AD and that almost two thirds of cases are female (Moustafa, 2021; Zhu et al., 2021). This sex difference has prompted an increased interest in sex-specific risk factors that might help to explain this difference (Chiow and Cicero, 2025). The cumulative effects of adverse childhood experiences (ACEs) such as trauma and chronic stress on cognitive health across the life span is an emerging focus of research (Anda et al., 2005; Heim and Nemeroff, 2001).

Physical, emotional, sexual abuse, and multiple types of neglect and household dysfunction have been linked to a wide variety of health issues as an adult, including mental disorders, cardiovascular disease, and neurodegenerative diseases like AD (Graves et al., 2021). Especially childhood trauma can have a significant impact on memory and cognitive capacity in later life (De Marco et al., 2017; Prieto et al., 2023). Critically, women experience ACEs more than men and are thus potentially more vulnerable to the long-term cognitive effects of ACEs (Madigan et al., 2023; Moustafa, 2021).

In addition to childhood stressors, women are also at higher risk of experiencing chronic social stressors such as for example caregiving responsibilities, gendered social expectations, and gender based violence. These can add to early life trauma and can also further lower cognitive resilience (Fliege et al., 2005; Haahr-Pedersen et al., 2020; Patel and Oremus, 2023). It is believed that these stressors act partially through the dys-regulation of the hypothalamic-pituitary-adrenal (HPA) axis and related stress-response mechanisms that play a role in depression and neurodegeneration (Heim and Nemeroff, 2001; Lei et al., 2025; Graves et al., 2021; Prieto et al., 2023). This combination of biological and social vulnerabilities could render women a population in which early adversity may have lasting effects on cognitive aging. This is a premise that has been increasingly supported by social and structural determinants of brain health studies (Livingston et al., 2020; Safiri et al., 2024).

Women’s elevated AD risk thus reflects the combined influence of biological, psychological, and sociocultural factors. Gendered caregiving responsibilities and associated sociocultural stressors in various cultural contexts have been linked to heightened chronic stress and depressive symptoms, both of which are independent risk factors for accelerated cognitive decline (Moustafa, 2021; Safiri et al., 2024). In Asia, especially in the India and China, cultural gender roles and family systems also play a role in the increased rate of emotional and physical neglect in women that can only have life-long consequences on cognitive abilities (Jain et al., 2022; Xiang et al., 2022). In the Middle East, domestic violence is another trauma that women are likely to experience, because gender inequality and violence are more common in this region. Besides, gender inequality, domestic violence, and emotional neglect documented across European, Asian, and Middle Eastern settings may each contribute to neurodegenerative risk through accumulated adversity (Haahr-Pedersen et al., 2020; Umberson and Karas Montez, 2010).

Situating ACEs within broader theoretical frameworks offers additional explanatory power for understanding their long-term cognitive consequences. Similarly, the social and structural determinants of brain health framework highlights systemic inequities. For example; how including poverty, housing instability, discrimination, and limited educational opportunity create conditions in which ACEs are not only more likely to occur but also more difficult to recover from Livingston et al. (2020), Röhr et al. (2022), Adkins-Jackson et al. (2023), Shaaban et al. (2026). Recognizing ACEs as modifiable risk factors embedded within these broader structural conditions is therefore essential for designing public health interventions that address not only individual trauma histories but also the social environments that generate and sustain them.

### 1.1 Adverse childhood experiences (ACEs) and Alzheimer’s disease

The empirical case for ACEs as drivers of AD risk has grown considerably in recent years. A meta-analysis of available studies estimated that individuals with a history of ACEs carry approximately 1.35 times the risk of developing AD compared to those without such experiences (Abouelmagd et al., 2024). This is also supported with longitudinal and retrospective cohort studies across diverse populations (Corney et al., 2022; He and Yang, 2024; Radford et al., 2017). Critically, this risk follows a dose-response pattern. The greater cumulative ACE burden is associated with steeper cognitive decline and earlier onset of dementia-related markers in later life (Hayes-Larson et al., 2025; Xiang et al., 2022). Not all ACE types, however, carry equivalent weight. Emotional neglect and sexual abuse, in particular, have been linked to more pronounced impairments. In adults, these impairments are related to memory and executive functioning and could involve structural changes in stress-sensitive neural circuits as potential mediating factors (De Marco et al., 2017; Fleck et al., 2025; Huang et al., 2023; Heim and Nemeroff, 2001). Early trauma also increases the likelihood of depression, anxiety and social withdrawal, which are risk factors on their own for cognitive decline and dementia. Thus it generates cumulative pathways of psychological processes that lead from adversity to neurodegeneration (Brown et al., 2021; Dafsari and Jessen, 2020; Prieto et al., 2023). There is also some evidence that early life adversity can be a contributor to both the process of amyloid accumulation and also the overall process of neurodegeneration in susceptible individuals, indicating a biological pathway as well as a psychological pathway to AD risk (Abo Hamza et al., 2023; Rose et al., 2025). Trauma experiences as measured by the five subscales of the Childhood Trauma Questionnaire represent individual level ACEs and are not fully understood as capturing broader structural or socioeconomic adversity, which is a limitation of the current measure of trauma.

### 1.2 Cognitive vulnerability in women

What makes women particularly susceptible to ACE-related cognitive decline goes beyond differential exposure rates. Inconsistent and/or intrusive parental behavior and sexual abuse have been shown to lead to higher levels of memory and executive functioning deficits among women compared to men who have been exposed to similar behaviors. This indicates a differential sensitivity to early adverse events for the sexes, and this could be due to the biological and socially learned differences in how stress is processed (Graves et al., 2021; Haahr-Pedersen et al., 2020; Lian et al., 2024). Psychosocially, less access to cognitively stimulating opportunities, chronic symptoms of depression associated with the persistent discrimination, and less access to health care in low-resourced environments. This is added to these early life risks, thus adding up vulnerabilities throughout the life course (Graves et al., 2021; Kim et al., 2025; Safiri et al., 2024; Umberson and Karas Montez, 2010).

Psychosocially, reduced opportunities for cognitive stimulation, chronic depressive symptoms linked to ongoing discrimination, and limited access to healthcare in lower-resource settings further compound these biological risks, creating layered vulnerabilities that accumulate across the lifespan (Graves et al., 2021; Kim et al., 2025; Safiri et al., 2024; Umberson and Karas Montez, 2010).

### 1.3 Current study

Despite growing interest in the role of early trauma in AD risk, relatively few studies have directly examined gender-specific patterns in the relationship between ACEs and cognitive outcomes in later life (Huang et al., 2023; Patel and Oremus, 2023). Despite the potential for various forms of ACEs to have different impacts on cognitive health (De Marco et al., 2017; Huang et al., 2023; Xiang et al., 2022), very few investigations have distinguished between these types of ACEs. In addition, previous studies have mostly concentrated either on subjective cognitive complaints or on general demential status rather than with clinical staging (GDS) and objective cognitive screening (MoCA).

The present study aims to address these gaps by examining the association between specific domains of childhood trauma (as measured by the CTQ) and both cognitive performance (MoCA) and clinical disease staging (GDS) in a sample of middle-aged and older women. Cognitive vulnerability in later life is defined operationally in this study as measurable cognitive decline (operationalized as MoCA performance below normal) and progression of clinical staging (operationalized as higher GDS score). This represents a range of cognitive risk states from sub clinical impairment to early dementia. In the general literature, these types of trajectories have been linked to progression to Alzheimer’s disease (Abouelmagd et al., 2024; Corney et al., 2022; Nasreddine et al., 2005; Reisberg et al., 1982). The present study is based on cognitive screening and clinical staging, therefore cannot directly determine the risk of AD with the help of AD-specific biomarkers and longitudinal follow-up. We focus particularly on the roles of emotional and physical neglect, given emerging evidence that neglect may be especially harmful for long-term neurocognitive development (Madigan et al., 2023; Zhu et al., 2025). We hypothesized that higher levels of childhood trauma, especially emotional and physical neglect, would be associated with poorer MoCA scores and more advanced GDS stages, even after accounting for age.

## 2 Materials and methods

### 2.1 Study design

This was a cross sectional study to explore the relationship between childhood trauma and cognitive functioning and cognitive vulnerability in women at a later point in life. The study was carried out on patients selected from two departments, Psychiatry and General Medicine at Tanta University Hospital, Egypt. A total of 55 women aged 58–79 years were included, ranging from no cognitive impairment to mild cognitive impairment (MCI) and early dementia (mild AD and other dementias).

The exclusion criteria were: (a) severe psychiatric disorders (e.g., schizophrenia), (b) neurological disorders other than dementia (e.g., major stroke, traumatic brain injury), and (c) inability to give informed consent. A battery of standardized questionnaires and clinical assessments were performed on all participants.

Each of the three assessment instruments used in this study had a different method of administration, appropriate to the specific instrument. All trained clinicians in Psychiatry and General Medicine Departments of Tanta University Hospital had previous experience in Psychiatric Hospitals in administering both GDS and MoCA. The CTQ is a self-report instrument, however, that has been validated and was independently completed by participants under standardized conditions.

### 2.2 Participants

The mean age of participants was 64.96 years (SD = 3.97), consistent with an age group at elevated risk for cognitive decline and AD. Participants were selected to represent a range of cognitive statuses, including women with normal cognition, MCI, and mild dementia (primarily early-stage AD). Demographic information about the participants in this study is reported in Table 1.

**TABLE 1:** Demographic information of patients.

Variable	Category	Percentage (%)	Mean (SD)
Age	58–79 years		64.96 (3.97)
Job status	Housewives	45.5%	
	Retired	16.4%	
	Freelancers	18.2%	
	Employees	20%	
Marital status	Married	45.5%	
	Widowed	27.3%	
	Divorced	16.4%	
	Not married	10.9%	
Education level	Uneducated	1.8%	
	Basic literacy	7.3%	
	Elementary school	9.1%	
	Preparatory school	18.2%	
	High school	30.9%	
	Bachelor’s degree	23.6%	
	Master’s degree	9.1%	
Number of children	0–6		3.07 (1.4)

In respect to their occupation, the sample mainly consisted of housewives. The notable percentage of housewives is interesting because socioeconomic pressures such as financial dependency and social isolation are likely to affect mental health and cognitive resilience in this group. In terms of marital status, 45.5% of the women were married. Marital status can also proxy other aspects of social support, such as emotional or practical support, which may be protective for brain health, and loneliness or stress in the event of widowhood or divorce (Umberson and Karas Montez, 2010).

The participants higher education is also an important demographic parameter. This is correlated with increased cognitive reserve and can delay the onset of AD clinical manifestations (Lian et al., 2024; Patel and Oremus, 2023). Participants age is also very important. In some cultures, the number of children may relate to higher caregiving burden and/or psychosocial stress, which might affect cognitive health (Graves et al., 2021; Haahr-Pedersen et al., 2020). The demographic attributes of the sample are summarized in Table 1.

### 2.3 Measures

#### 2.3.1 Childhood Trauma Questionnaire (CTQ)

Childhood trauma was measured using the well-validated and widely-used 28 item Childhood Trauma Questionnaire (CTQ; Bernstein et al., 2003) self report form. The CTQ has five clinical subscales: Emotional Abuse; Physical Abuse; Sexual Abuse; Physical Neglect; and Emotional Neglect. For each item, a 5 point Likert scale from 1 (Never true) to 5 (Very often true) is used. The subscale scores provide an indication of the severity of each trauma domain. The CTQ also features a Minimization/Denial scale to assess for under reporting of trauma. The scores for the trauma subscales increase the higher the adversity experienced. The CTQ was self-report and each participant completed it on their own. The CTQ is administered in this way regularly, and it uses this method in its validation process (Bernstein et al., 2003). The questionnaire was filled out in a quiet, private location and research staff were on hand to clarify any misunderstandings of items without influencing responses.

#### 2.3.2 Global Deterioration Scale (GDS)

Global cognitive decline and dementia staging were assessed with the Global Deterioration Scale (GDS) developed by Reisberg et al. (1982). The GDS classifies individuals into seven stages: Stage 1 (no cognitive decline), Stage 2 (very mild cognitive decline), Stage 3 (mild cognitive impairment), and Stages 4–7 (progressive stages of dementia from mild to very severe). In the present study, GDS scores were used to characterize the severity of cognitive impairment and to identify participants further along the spectrum of cognitive vulnerability in later life. Unlike the self-report CTQ, the GDS is a clinician-rated instrument requiring professional judgment about functional and cognitive status. In this study, GDS staging was conducted by a trained clinician with previous experience in administering the GDS.

#### 2.3.3 Montreal Cognitive Assessment (MoCA)

Cognitive performance was evaluated using the Egyptian Arabic version of Montreal Cognitive Assessment (MoCA), a brief screening tool designed to detect MCI and early dementia (Nasreddine et al., 2005). The MoCA assesses multiple cognitive domains, including attention, memory, visuospatial skills, language, executive function, abstraction, and orientation. Total scores range from 0 to 30, with scores ≥ 26 typically considered within the normal range. However, population-based normative studies in Arab adults suggest that culturally adjusted cutoffs typically in the range of 21–22 may be more appropriate for detecting MCI in non-Anglo-Saxon contexts (Amro et al., 2025; Rahman and El Gaafary, 2009). The lower scores reflect higher levels of cognitive impairment. For this study, MoCA scores were applied as a continuous assessment of global cognition and to classify patients as having MCI or early dementia. MoCA was conducted in a structured face-to-face manner with standardized administration protocol as described by Nasreddine et al. (2005) by a trained clinician. In the same way as the GDS, the same administrator administered all MoCA assessments, and thus all assessments were administered and scored in a consistent manner. Moreover, the MoCA is not a self-report measure like the CTQ and its structured format is intended to reduce examiner variability.

This study included both the MoCA and the GDS. The MoCA provides a quantitative, domain-specific assessment of cognitive performance making it sensitive to subtle early-stage impairments and allowing for fine-grained differentiation across participants (Nasreddine et al., 2005). However, the GDS is a clinician rated global staging instrument that provides a staging system for the whole spectrum of dementia from normal aging to severe functional loss with behaviorally defined presentations (Reisberg et al., 1982). These instruments give two different, but complementary, views into cognitive vulnerability. The MoCA is an objective measure of performance on standard tasks, and the GDS is a measure of actual clinical severity. Both enable the study to determine if childhood trauma is associated with cognitive dysfunction as well as clinical deterioration.

### 2.4 Data analysis

Data were analyzed using IBM SPSS Statistics. Preliminary analyses included descriptive statistics for all variables and assessment of normality for MoCA and GDS scores using the Shapiro–Wilk test. Both GDS (*p* < 0.001) and MoCA (*p* = 0.024) deviated significantly from normal distributions, justifying the use of non-parametric methods for correlation analyses.

A Spearman rank order correlation (ρ) was performed between GDS and MoCA scores to investigate the correlation between the 2 variables for the overall clinical staging. MoCA scores were also correlated with CTQ subscale scores using non-parametric methods to examine relationships between type of trauma and cognitive functioning.

To evaluate the unique contributions of each CTQ subscale to MoCA and GDS scores, controlling for age, multiple linear regression (MLR) models were then estimated. All CTQ subscales (Physical Abuse, Emotional Abuse, Sexual Abuse, Physical Neglect, Emotional Neglect) were included as predictors with MoCA and GDS separately analyzed. R squared (R^2^) was used to assess model fit, and F tests were used to assess the statistical significance. GDS is ordinal, but linear regression was employed as an approximation which is noted as a limitation.

### 2.5 Transparency and openness

All measures, participant characteristics, inclusion criteria, and statistical analyses conducted in this study are fully reported. The sample size of *N* = 55 was determined by participant availability during the recruitment period. A *post hoc* power analysis was performed to check the statistical power of this sample for the use in the primary analyses with GPower 3.1. The observed R^2^ in the multiple regression model predicting MoCA scores is 0.725, which is considered a large effect size (f^2^ = 2.636). With six CTQ predictors and an α of 0.05, this results in an estimated power of > 0.99, which is a well-powered sample for detecting the effects observed.

Due to ethical and legal considerations related to participant confidentiality, the de-identified data underlying the reported findings are not publicly available. The study received institutional ethics approval and was carried from May 2025 to December 2025 (Ethical Research Committee code is 36264PR925/5/24).

The analytic code used for the statistical analysis (IBM SPSS Statistics) is not publicly available because of institutional data governance policies but all analytic procedures and software versions have been described in detail in the Method section.

Study materials are not publicly available due to being standardized, copyrighted instruments such as the Montreal Cognitive Assessment, Global Deterioration Scale and Childhood Trauma Questionnaire. There was no pre-registration of the study design and analytic plan.

## 3 Results

### 3.1 Global Deterioration Scale (GDS)

The Global Deterioration Scale (GDS) is an established tool for assessing stages of cognitive decline in individuals with Alzheimer’s disease (AD). The scale ranges from Stage 1 (no cognitive decline) to Stage 7 (very severe cognitive decline). Stages 1–3 represent pre-dementia stages, whereas Stages 4–7 represent dementia stages.

The GDS scores of 55 women were distributed as illustrated in Figure 1 and their mean ages. The majority of participants were in Stage 3 (Mild Cognitive Impairment – MCI) and a smaller proportion were in Stage 4 (Early Confusional/Early Dementia). This trend suggests that mostly women in the early stages of cognitive decline, without severe impairment, make up the sample. The mean GDS score is 3.24, which is consistent with this distribution and confirms that the majority of subjects have from mild cognitive impairment, a clinical state which is often linked to a higher risk of progressing to Alzheimer’s disease.

**FIGURE 1:**
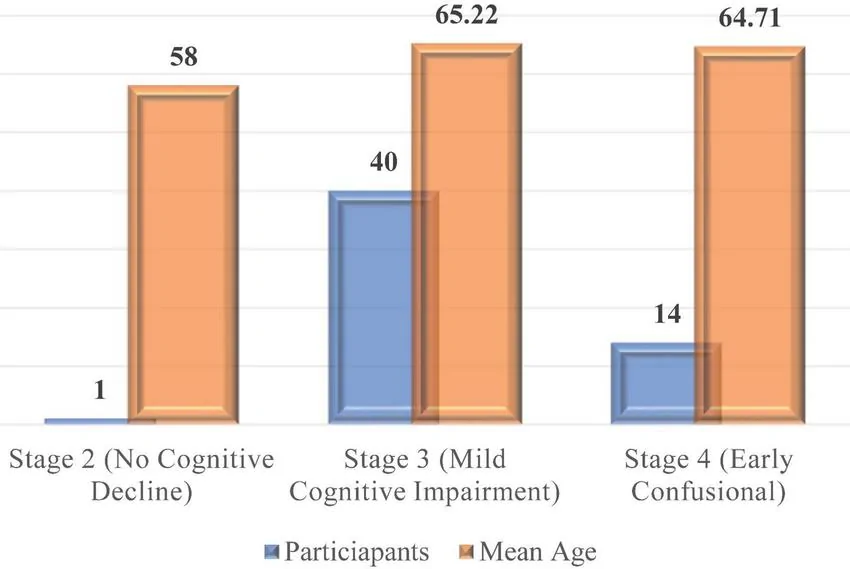
Sample distribution for Global Deterioration Scale survey.

### 3.2 Montreal Cognitive Assessment (MoCA)

The Montreal Cognitive Assessment (MoCA) scores were analysed to better understand the relationship between childhood trauma, stress, and cognitive health. The overall distribution of MoCA scores is presented in Figure 2.

**FIGURE 2:**
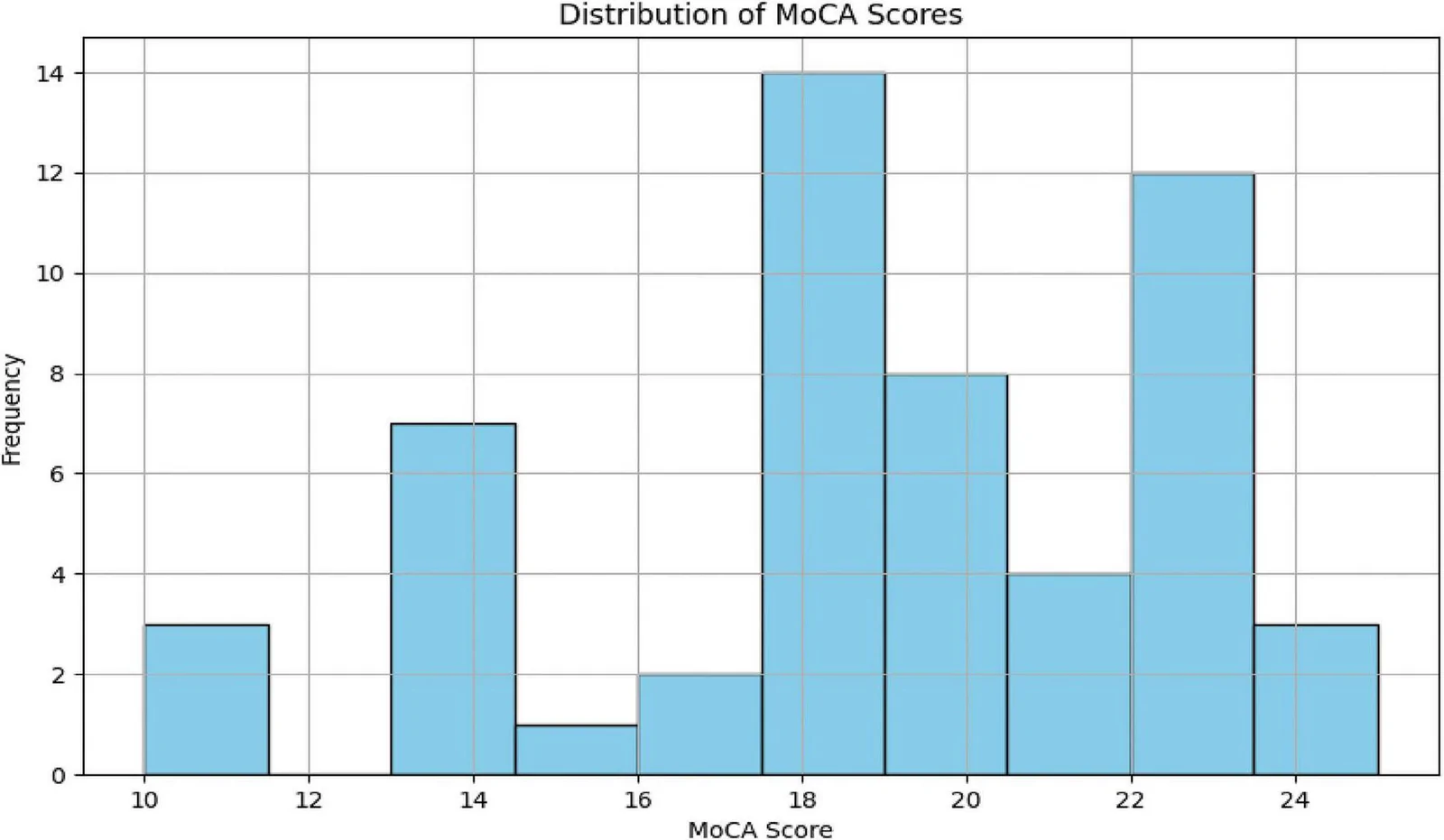
Distribution of MoCA score.

The mean MoCA score was 18.67 (SD = 3.70), below the customary score of 24 or more that is considered to indicate a normal cognitive function. Results of less than 26 can indicate mild cognitive impairment (MCI). The mean MoCA score of 18.67 suggests that, on the average, the participants are likely to be suffering from the early stages of cognitive impairment and thus can be considered to be at a higher risk for having Alzheimer’s disease.

The 25th percentile was 18; the median was 18.5 and the 75th percentile was 22. This means that a majority of the participants had a score of 18–2 which falls in the range of mild cognitive impairment. Total scores varied from 10 (signifying greater impairment) to 25 (approaching normal cognitive functioning), with significant inter-individual variation in cognitive status.

The best score on MoCA is found as 25, which indicates that some of the participants are doing relatively well on the cognitive assessment. A score of 10 or less is in the lowest 25 percent, which means at least one participant had moderate cognitive dysfunction, typical of higher levels of neurocognitive disorder. The standard deviation is 3.70, which is relatively high, indicating a lot of variation in cognitive performance. This is to be predicted as participants were recruited at various stages of cognitive health including women with normal cognition and women with MCI. Memory problems are an important point to note as they are a common prodrome to AD, but they are not enough to fully diagnose AD on their own.

The MoCA findings are especially salient with respect to understanding how early life stress and childhood trauma (as assessed by the CTQ) relate to cognitive health in adulthood. This pattern is consistent with childhood trauma exposure having persistent impacts on cognition as most of the children in the study fall within the MCI range of scores. Structural and functional changes in brain areas related to memory and learning (e.g., hippocampus) have been linked to early trauma, which may result in cognitive problems later in life. These cross sectional data suggest that early adversity may hasten the process of cognitive decline, particularly for women with a genetic predisposition to Alzheimer’s disease or who have other risk factors, but do not prove this.

### 3.3 Childhood Trauma Questionnaire (CTQ)

To examine the effects of childhood trauma on cognitive well-being in adulthood, and specifically the risk of AD, scores on the Childhood Trauma Questionnaire (CTQ) subscales were analyzed.

The highest mean score was on the Emotional Abuse subscale which was 16.82 (SD = 1.27). This means that majority of participants experienced moderate to severe emotional abuse during childhood. Emotional abuse can involve acts like verbal abuse, embarrassment or a constant lack of concern for emotional needs. These experiences have been linked to negative cognitive and emotional consequences, such as memory loss and a higher risk of AD (Hedges et al., 2024; Joshi and Yeo, 2025; Li et al., 2025). These results indicate that emotional abuse is a likely important factor affecting future cognitive outcomes.

The Physical Abuse mean score was 14.98 (SD = 2.22) suggesting that many participants had experienced physical abuse to different extents. The standard deviation is quite large relative to the mean score, indicating that there is a large spread of physical abuse experiences, ranging from mild to more serious.

The mean score for the Sexual Abuse subscale was 13.09 (SD = 2.27) indicating that sexual abuse was similarly relatively common in participants with varying severity. There is considerable variation in experiences, including sexual harassment, sexual assault, or sexual rape as measured by the standard deviation. Sexual abuse has extreme consequences on mental health and brain function, and often results in emotional regulation issues and cognitive difficulties (Graves et al., 2021). The correlation and regression analyses below explore further the relationship between sexual abuse and the severity of clinical disease in the present study.

The mean score for Physical Neglect was 14.98 (SD = 2.22), suggesting that participants had moderate levels of physical neglect (e.g., poor food, shelter, and medical care).

The mean for the Emotional Neglect subscale was 14.91 (SD = 1.46), indicating relatively high levels of emotional neglect (a pattern of persistently failing to satisfy a child’s emotional needs or provide nurturing and affection). The high standard deviation for this subscale suggests a great variation of emotional neglect experiences among the participants.

Finally, the Minimization/Denial subscale, which measures the tendency to downplay or deny traumatic experiences, had a mean score of 0.00 and a standard deviation of 0.00. This indicates that none of the participants endorsed items suggestive of minimization or denial of trauma. The low minimization/denial scores observed in this sample suggest that participants did not exhibit a significant positive response bias in reporting their childhood experiences. It is therefore supporting the validity of the CTQ trauma disclosures. The CTQ Minimization-Denial subscale was designed specifically to detect underreporting tendencies.

Figures 3–5 presents the distribution of CTQ subscale scores and total trauma burden across all participants. As shown in Figure 3, scores on all five subscales fell predominantly in the moderate-to-severe range, with Emotional Neglect recording the highest median score (Median = 17.0, IQR = 16.0–17.0) and Physical Abuse the lowest (Median = 12.0, IQR = 12.0–13.0).

**FIGURE 3:**
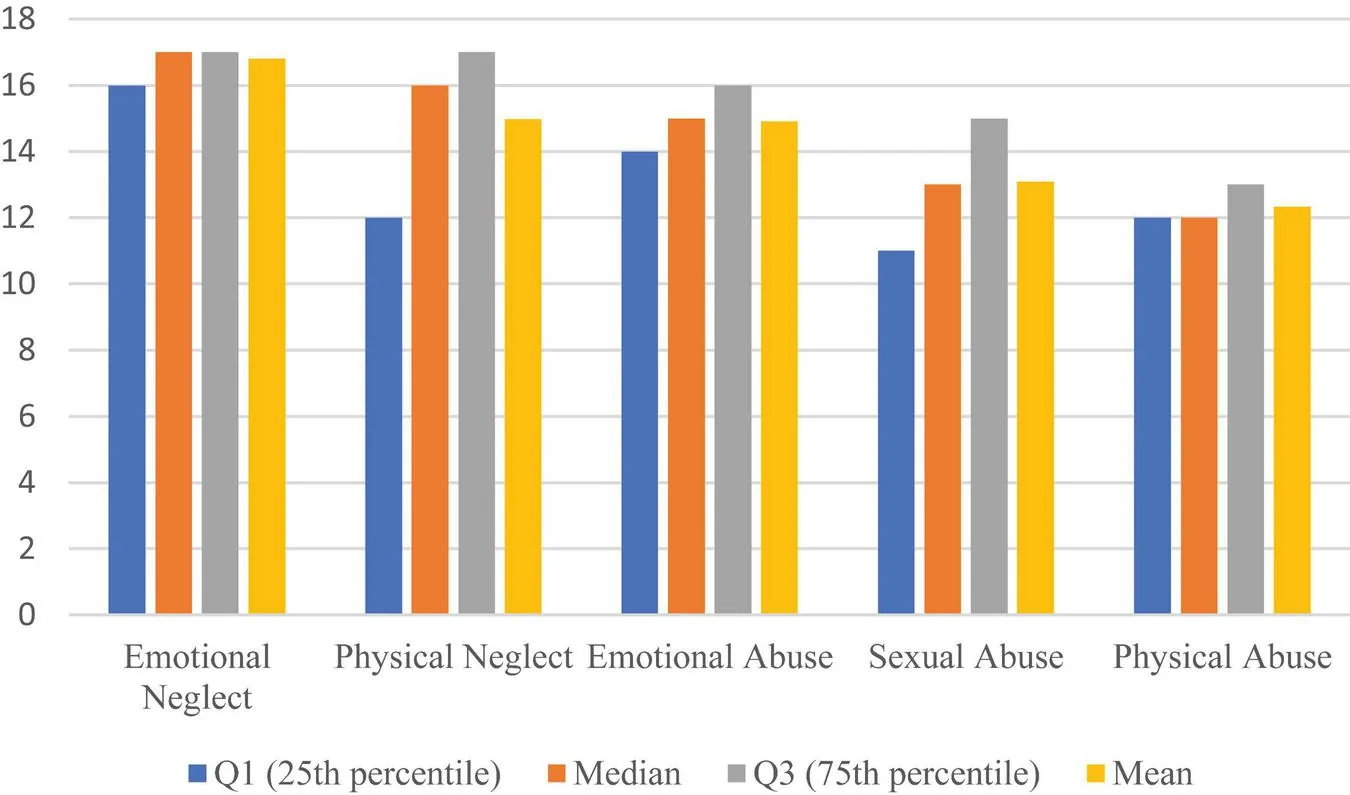
CTQ subscale score distribution (median, Q1, Q3).

**FIGURE 4:**
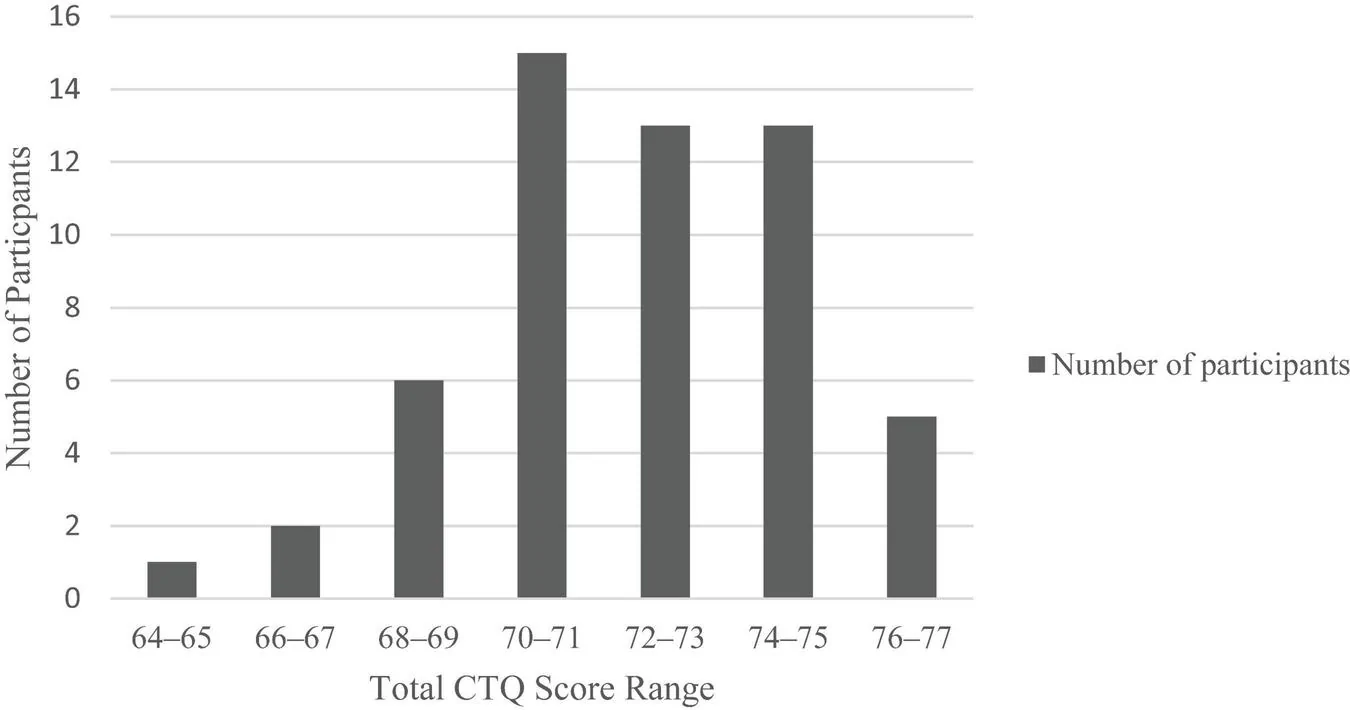
Total CTQ score distribution.

**FIGURE 5:**
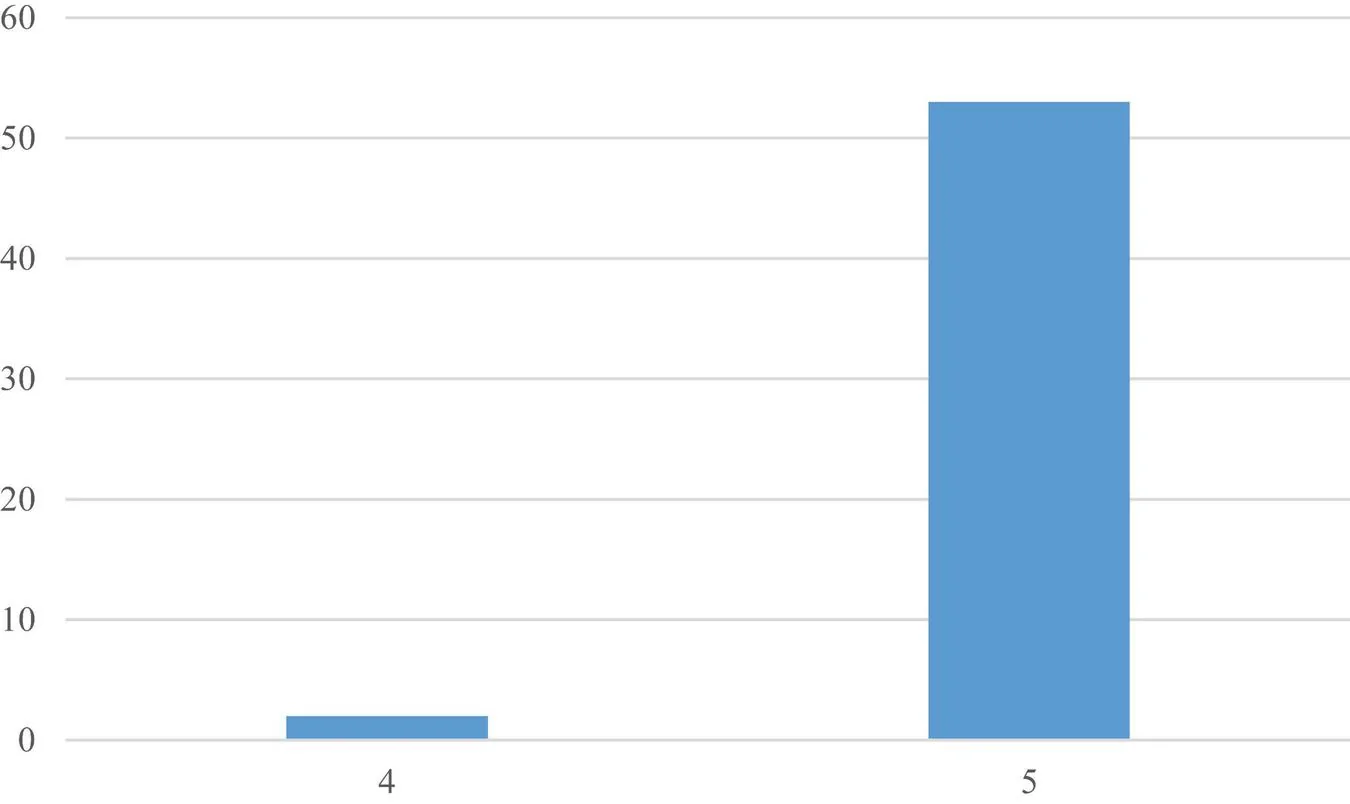
ACE co-occurrence (no. of ACE types above moderate threshold).

Total CTQ scores ranged narrowly from 65 to 78 (M = 72.11, SD = 2.76) as showing in Figure 4. The findings are indicating that the sample as a whole experienced elevated and broadly comparable levels of cumulative trauma.

Figure 5 demonstrates that 53 of 55 participants (96.4%) scored above the moderate-severity threshold on all five ACE subscales simultaneously. The remaining two participants exceeded the threshold on four of five subscales.

### 3.4 Correlation between GDS and MoCA scores

Before conducting the primary analyses, the distributional properties of the variables were examined. The Shapiro–Wilk test was used to assess normality, and results indicated that both GDS scores (*p* < 0.001) and MoCA scores (*p* = 0.024) significantly deviated from a normal distribution. Consequently, a non-parametric Spearman’s rank-order correlation (ρ) was used to investigate the relationship between clinical disease staging and objective cognitive performance.

There was a strong negative correlation between GDS stages and MoCA scores with the bivariate Spearman correlation, ρ (53) = −0.503, *p* < 0.001. This relationship is shown in Figure 6. This statistically significant negative correlation suggests that the more clinically deteriorated the subjects are (i.e., the higher the GDS stage), the poorer their cognitive performance (i.e., the lower the MoCA score). These results indicate that there is significant overlap in the clinician observed functional decline and the psychometric assessment of cognition in this sample of women, lending support for concurrent validity of GDS and MoCA in this sample.

**FIGURE 6:**
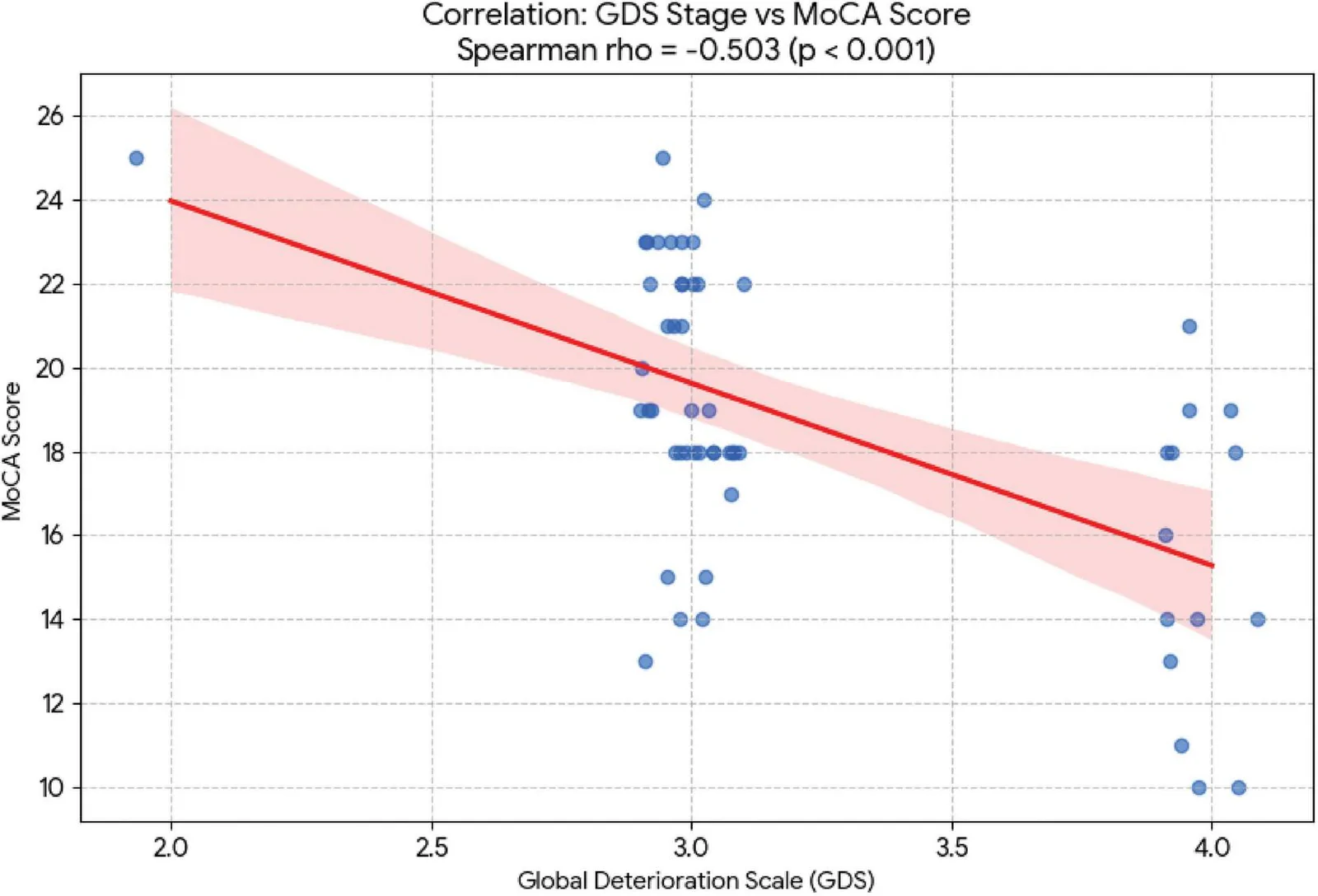
Correlational analysis of MoCA score and GDS.

### 3.5 Correlations between MoCA and GDS with CTQ subscales

Pearson correlations, corrected for multiple comparisons using the Benjamini-Hochberg FDR procedure (primary) and Bonferroni correction (sensitivity check) were computed to examine associations between both MoCA scores and GDS stages, see Table 2. These analyses shed light on how specific types of childhood trauma relate to adult cognitive performance and clinical disease staging.

**TABLE 2:** Correlation matrix between MoCA scores and GDS with CTQ subscales.

CTQ subscale	Outcome	*r*	*p* (uncorrected)	*p* (FDR-BH)	*p* (Bonferroni)	Significant (FDR)?*	Significant (Bonf)?*
Emotional neglect	MoCA	-0.85	< 0.001	<0.001	< 0.001	Yes	Yes
Physical neglect	MoCA	-0.80	< 0.001	<0.001	< 0.001	Yes	Yes
Emotional abuse	MoCA	-0.65	< 0.001	<0.001	< 0.001	Yes	Yes
Physical abuse	MoCA	-0.50	< 0.001	<0.001	0.001	Yes	Yes
Sexual abuse	MoCA	-0.28	0.038	0.038	0.384	Yes	**No**
Emotional neglect	GDS	0.85	< 0.001	<0.001	< 0.001	Yes	Yes
Physical neglect	GDS	0.80	< 0.001	<0.001	< 0.001	Yes	Yes
Emotional abuse	GDS	0.65	< 0.001	<0.001	< 0.001	Yes	Yes
Physical abuse	GDS	0.50	< 0.001	<0.001	0.001	Yes	Yes
Sexual abuse	GDS	0.28	0.038	0.038	0.384	Yes	**No**

*FDR-BH, Benjamini-Hochberg false discovery rate correction; Bonf, Bonferroni familywise error rate correction; corrected significance threshold for Bonferroni: α = 0.005 (10 comparisons). *N* = 55.

However, emotional abuse was highly negatively correlated with MoCA scores (*r* = −0.65, *p* < 0.01). This indicates that a greater amount of emotional abuse correlated with poorer cognitive functioning. A parallel positive correlation was found with GDS staging (*r* = 0.65, *p* < 0.001), suggesting that more advanced clinical deterioration was also associated with greater history of emotional abuse. When considered together, these findings indicate that emotional abuse has a constant effect both on objective cognitive functioning and disease severity as rated by clinicians. This aligns with previous studies, which showed that emotional abuse is associated with subsequent memory and executive function impairments (Madigan et al., 2023; Heim and Nemeroff, 2001).

MoCA had a moderate negative correlation with Physical Abuse (*r* = −0.50, *p* < 0.05). This suggests that physical maltreatment also is correlated with cognitive functioning in adulthood. But the association does not seem to be as strong as for neglect. In a similar fashion, Physical Abuse was positively associated with GDS stage (*r* = 0.50, *p* < 0.001). This suggests that higher levels of physical abuse history were associated with higher clinical levels of impairment. This finding was robust to FDR and Bonferroni corrections (p_corrected = 0.001) for both MoCA and GDS associations. This is consistent with evidence suggesting that early physical trauma can lead to neurocognitive deficits via chronic stress and inflammatory processes (Li et al., 2025; Majer et al., 2010).

The correlation between sexual abuse and cognitive outcomes MoCA scores reached conventional significance prior to correction (*r* = −0.28, *p* = 0.038). A comparable pattern, in the theoretically expected positive direction, was observed for GDS (*r* = 0.28, *p* = 0.038). However, both associations did not survive the conservative Bonferroni correction (p_corrected = 0.384). Under the less conservative but more appropriate FDR-BH correction, the associations remained statistically significant (p_FDR = 0.038). This suggests a weak but potentially meaningful relationship that warrants replication in larger samples. All four other subscales correlated with both MoCA and GDS survived both correction procedures. This further suggests that sexual victimization may be associated with cognitive impairment and advancing disease stage, but these effects may be partly mediated by intervening factors such as post-traumatic stress disorder (PTSD) or depressive symptoms (Burri et al., 2013; Moustafa, 2021; O’Neal, 2023).

Physical Neglect had a strong negative correlation with MoCA (*r* = −0.80, *p* < 0.01). Such findings are indicating that higher levels of physical neglect were strongly associated with poorer cognitive performance. Correspondingly, Physical Neglect showed an equally strong positive correlation with GDS stage (*r* = 0.80, *p* < 0.001). This suggest that greater physical neglect was associated with more severe clinical staging of cognitive decline. Emotional Neglect showed an even stronger negative correlation with MoCA (*r* = −0.85, *p* < 0.01) and an equally strong positive correlation with GDS (*r* = 0.85, *p* < 0.001) which represents the strongest associations observed across all subscales and both outcomes. These findings suggest that neglect (particularly emotional neglect) may be especially detrimental to long-term cognitive health. This is consistent with prior studies linking neglect to hippocampal alterations and cognitive decline (Madigan et al., 2023; Zhu et al., 2025). Across all subscale-outcome pairs, the magnitude and direction of correlations were strikingly consistent between MoCA and GDS, indicating that childhood trauma, and neglect in particular predicts not only poorer cognitive test performance but also greater clinical severity of neurocognitive deterioration.

### 3.6 Multiple regression analyses: childhood trauma, MoCA, and GDS

To further examine the impact of childhood trauma on cognitive performance and clinical staging, multiple linear regression analyses were conducted with CTQ subscales as predictors and MoCA and GDS scores as outcomes. Age was included as a covariate.

The joint effects of the CTQ subscales and age accounted for 72.5% of the variance (R^2^ = 0.725, *p* < 0.01) in cognitive performance for the MoCA model. For the GDS model, the same predictors explained 50% of the variance in cognitive decline (R^2^ = 0.50, *p* < 0.05). Since MoCA (range: 0–30) and GDS (range: 1–7) use different scales, it is expected that regression coefficients differ between the two models. This scale difference is not a reflection of weaker substantive relationships, but smaller absolute GDS coefficients. The regression coefficients are given in Table 3.

**TABLE 3:** Multiple linear regression analysis for MoCA and GDS scores.

Predictor variable	Coefficient (MoCA, β)	Standard error (MoCA)	Coefficient (GDS, β)	Standard error (GDS)
Constant	5.9224	2.471	0.7533	0.314
Age	−0.0026	0.018	−0.0003	0.002
Physical abuse	−0.0787	0.052	0.0100	0.007
Emotional abuse	−0.0212	0.045	0.0027	0.006
Sexual abuse	−0.0184	0.037	0.0023	0.005
Physical neglect	−0.0100	0.038	0.0013	0.005
Emotional neglect	−0.0877	0.066	0.0112	0.008

Emotional Neglect was the most powerful predictor variable in both models. The opposite orientation of MoCA (β = −0.0877) and GDS (β = 0.0112) is shown. These findings suggest that more emotional neglect was related to worse cognitive functioning and more severe clinical staging, controlling for age and other trauma types.

Physical Neglect also had a negative coefficient for MoCA (β = −0.0100) and a positive coefficient for GDS (β = 0.0013). These are pointing toward higher levels of physical neglect being related to poorer cognitive performance on both measures. Physical Abuse was moderately negatively associated with MoCA (β = −0.0787) and was strongly positively associated with GDS (β = 0.0100). This outcome is based on scale differences between outcomes and indicates some specificity of physical abuse effects with regards to performance based cognitive screening versus clinical staging.

After controlling for other trauma types and age, there were no significant independent effects of Sexual Abuse on MoCA (β = −0.0184) or GDS (β = 0.0023); suggesting that the effect of sexual abuse on cognition may be partially mediated by psychological factors such as PTSD or depression, which were not directly measured in this study (Burri et al., 2013; Flatt et al., 2017; Prieto et al., 2023).

In these models, age was not a significant predictor of either MoCA (β = −0.0026) or GDS (β = −0.0003), indicating that in this relatively small age window, early life trauma may account for more variance in cognitive outcomes than age alone.

### 3.7 Model diagnostics

To assess multicollinearity among the correlated CTQ subscales, variance inflation factors (VIFs) were calculated for all predictors in both regression models. VIFs ranged from 1.07 to 1.67 (age = 1.15; physical abuse = 1.43; emotional abuse = 1.07; sexual abuse = 1.66; physical neglect = 1.67; emotional neglect = 1.51), well below conventional thresholds of concern (VIF > 5–10). This means that there was no high degree of multicollinearity among the CTQ subscales, but the CTQ subscales were correlated with each other. Regarding residual assumptions, we note that GDS is an ordinal outcome analyzed here via linear regression, a choice that affects the distributional assumptions underlying ordinary least squares. Ordinal or generalized linear modeling approaches would be a more statistically appropriate choice for this outcome in future work.

## 4 Discussion

This empirical study examined associations between specific types of childhood trauma and cognitive functioning in adulthood, with a focus on women’s cognitive vulnerability in later life. Using both a clinical staging measure (GDS) and an objective cognitive screening tool (MoCA), we found that neglect, particularly emotional neglect, showed the strongest associations with cognitive impairment and clinical decline in later life. Notably, the pattern of associations between CTQ subscales and MoCA scores was strikingly consistent with that observed for GDS staging. This shows strong correlation coefficients across CTQ and GDS with each subscale. This convergence across two conceptually distinct instruments strengthens confidence in the robustness of these findings and suggests that childhood trauma influences not only cognitive test performance but also the broader clinical trajectory of neurocognitive deterioration.

The results showed very strong negative correlations between emotional neglect and MoCA, and strong negative correlations between physical neglect and MoCA, meaning that those with higher scores for neglect were more likely to be more cognitively impaired on standardized cognitive screening and to be rated as more advanced in the clinical progression of their disease. Both associations were significant after FDR and Bonferroni corrections, confirming their statistical significance. Emotional neglect was the strongest multilinear predictor of MoCA and GDS scores in the multivariable regression models. Based on these results there is a possibility that neglect, particularly the lack of emotional support and nurturance, can have long-lasting implications on neurocognitive development and resilience. These findings are consistent with earlier studies that show a link between neglect and long term cognitive deficits, and a faster cognitive decline (Majer et al., 2010; Naumova et al., 2019; Madigan et al., 2023; Zhu et al., 2025).

Similarly, there was a strong negative correlation with MoCA, suggesting that emotional maltreatment in childhood can impact cognitive functioning, possibly due to chronic stress system activation and changes in brain structure and function (Heim and Nemeroff, 2001; Huang et al., 2023). A similar association was found with the GDS staging which remained significant after FDR and bonferroni correction. The consistency of this double outcome indicates that emotional abuse has risk for both objectively measurable cognitive dysfunction and clinically relevant disease progression. Previous research has shown that emotional abuse is linked to lower levels of memory, executive function, emotional regulation and higher risk of mood and anxiety disorders (Hedges et al., 2024; Joshi and Yeo, 2025; Lei et al., 2025). Physical abuse demonstrated that early physical abuse is linked to cognitive outcomes but not as significantly as neglect. Similarly, there was moderate correlation (*r* = 0.50) in the expected direction (positive) with GDS stage. Both correction procedures led to an association effect that was still present (p_Bonferroni = 0.001). Convergent evidence from MoCA and GDS suggests that physical abuse is not negligible in the clinical spectrum of cognitive decline in this population but is smaller in magnitude than adversities experienced through neglect. This aligns with the wider literature on the association between physical abuse and cognitive impairment and dementia, which may be due to chronic stress, vascular risk and inflammation (Li et al., 2025; Patel and Oremus, 2023).

In the Sexual abuse subscale, PTSD, depression, and anxiety are known intermediate mechanisms between exposure to adversity and cognitive impairment, and are not necessarily related to sexual abuse alone (Günak et al., 2025; Lowry et al., 2022). This study finds that sexual abuse operates primarily through distal psychological pathways including PTSD, depression, and disrupted social functioning rather than through the more direct neurodevelopmental routes associated with early and sustained neglect (Burri et al., 2013; Flatt et al., 2017; O’Neal, 2023). However, the sexual abuse–cognition association might also be moderated by protective factors like psychological resilience, social support, and access to trauma-focused treatment, factors that were not assessed in the current study (Haahr-Pedersen et al., 2020; Radford et al., 2017).

It is also important that the sexual abuse–cognition association did not withstand the more stringent Bonferroni threshold (p_corrected = 0.384 for both MoCA and GDS). This finding of no significant difference after conservative correction is similar to what has been previously reported. This suggests that sexual abuse can have a more psychologically mediated influence on cognitive decline, and that it does not have a direct influence similar to those of the adversities associated with neglect (De Marco et al., 2017; Fleck et al., 2025).

Taken together, our results suggest that it is important to differentiate kinds of ACEs. Total trauma burden was moderately and negatively associated with cognition, but specific adversities especially emotional and physical neglect predicted cognitive outcomes more than did composite ACE scores. These same associations were seen across the range of MoCA performance and GDS clinical staging, further supporting the idea that adversities associated with neglect affect the entire spectrum of cognitive aging. This aligns with more recent research indicating that some ACEs (such as neglect, household dysfunction) are more strongly linked to late life cognitive impairment and dementia than others (Corney et al., 2022; Xiang et al., 2022; Zhu et al., 2025).

There are some general interpretive points which deserve to be considered in the present investigation. In the current analyses, the CTQ subscales are examined independently, as realistically, ACEs seldom co-occur without emotional neglect. For example, multiple types of ACEs often go hand-in-hand with physical neglect and dysfunction within the household, and the effects of multiple ACEs may be synergistic and not just additive on cognition (Xiang et al., 2022). Additionally, it should be emphasized that the cross-sectional design of this study does not allow for interpretation as cause and effect. Causality can be inferred from the fact that childhood trauma occurs earlier in life than cognitive outcomes. Other variables such as depression severity, PTSD, sleep disturbance, inflammation, and lifestyle factors might significantly contribute to the associations found (Dafsari and Jessen, 2020; Li et al., 2025; Prieto et al., 2023).

Our results also support the broader literature on the long-term impact of early adversity on brain health and later-life cognitive vulnerability. These findings extend the broader ACE-AD literature, in which dysregulated stress responses and their downstream neurobiological consequences have been proposed as key mechanisms linking early adversity to accelerated cognitive aging (De Marco et al., 2017; Heim and Nemeroff, 2001; Huang et al., 2023; Lei et al., 2025).

These results are especially problematic from a gender point of view. Women are at unique risk for the cognitive effects of early adversity over the life course, as they have a combination of biological and psychosocial risk factors that can exacerbate these effects (Haahr-Pedersen et al., 2020; Madigan et al., 2023). They also have unique biological risk factors including differential effects of APOE4 (De Marco et al., 2017; Abo Hamza et al., 2023; O’Neal, 2023), and hormonal changes. The present study aligns with the emerging literature of the need for dementia risk assessment and prevention interventions that are trauma-informed and gender sensitive (Abouelmagd et al., 2024; Patel and Oremus, 2023; Safiri et al., 2024).

This study did not measure two biological factors, which, however, are important in interpreting the results of this study. Hormone changes that occur with menopause are a big change in neuroprotection. This is because as estrogen levels drop, it is believed that this makes one more vulnerable to the neurodegenerative mechanisms of AD (De Marco et al., 2017; Moustafa, 2021; O’Neal, 2023). Many of the subjects in the present sample were peri or post-menopausal due to the age group of the present sample (58–79 years). Second, the APOE4 genotype, a higher risk genotype for dementia in women than in men, may moderate the impact of ACE history, resulting in a higher risk of cognitive vulnerability in women carrying both factors (De Marco et al., 2017; Abo Hamza et al., 2023; Zhu et al., 2021). However, reproductive status and APOE4 were not evaluated in the present study; these factors could be included in future studies to investigate the interaction between biological predispositions and environmental exposures with regard to women’s trajectories in cognition.

Since we found that assessing childhood trauma, particularly neglect, is associated with identifying women at higher risk for cognitive decline later in life, this may be a valuable screen to conduct routinely. Interventions to decrease the impact of early life adverse events, including trauma, might be psychotherapy, improving social support and early screening for mood and anxiety disorders, which could improve long term cognitive health (Brown et al., 2021; Dafsari and Jessen, 2020; Joshi and Yeo, 2025).

This study has some limitations which should be noted. One, this is a cross sectional study, which means conclusions about the cause of the relationship cannot be drawn. We saw strong links between child abuse and cognitive outcomes, but could not conclude causality or direction of effects. Longitudinal studies are warranted to better understand a path from early adversity to cognitive decline and AD.

Second, only females, from one hospital in Egypt, were included in the sample and the sample size was relatively small (*N* = 55). This restricts the ability to make generalizations and to compare male and female. Future research should be conducted on both sexes and larger cohorts with greater diversity to investigate for possible sex differences in the trauma–cognition relationship (Chiow and Cicero, 2025; Patel and Oremus, 2023).

Thirdly, childhood trauma was measured retrospectively by self-report (CTQ). Retrospective reports can be affected by recall bias or mental state and the CTQ is well validated (Bernstein et al., 2003). Using other data sources (such as clinical records) and other objective stress markers (e.g., cortisol, inflammatory markers) may give a fuller picture of the biological processes by which trauma translates into cognitive outcomes (Heim and Nemeroff, 2001; Lei et al., 2025; Rose et al., 2025).

Fourth, GDS is an ordinal scale which was analysed as a linear scale. Statistically more appropriate would be ordinal logistic regression or other models that are specific for ordered outcomes. Analytic methods which better capture the measurement properties of GDS should be considered for future work.

Fifth, important potential mediators and moderators (depression, anxiety, PTSD symptoms, social support, APOE genotype, and lifestyle factors, such as physical activity, smoking and diet) were not measured. These variables would be valuable to incorporate into future models to help clarify how ACEs relate to cognitive pathways and the risk of AD (Dafsari and Jessen, 2020; Li et al., 2025; Prieto et al., 2023).

Sixth, some psychosocial factors were not assessed in the present study such as caregiving burden, exposure to gendered violence and socioeconomic stress. While these are not the main focus of the present study, the lack of these factors means that they cannot be excluded as potential confounder factors in the relationships between childhood trauma and the cognitive outcomes. Future research should include direct measurements of these variables to allow for statistical separation of the effects of childhood trauma from the effects of current psychosocial stressors, as well as to explore whether the variables mediate or moderate the trauma – cognition relationship.

## 5 Conclusion

This study examined the association between childhood trauma and cognitive impairment in a sample of middle-aged and older women. The findings indicate that early-life trauma particularly emotional and physical neglect is strongly associated with poorer cognitive performance (MoCA) and more advanced clinical staging (GDS) in adulthood. Emotional neglect emerged as the most powerful predictor of cognitive outcomes, even after controlling for age and other trauma types.

Taken together, these findings suggest that the effects of neglectful trauma have a long-lasting impact on brain function and might leave individuals vulnerable to cognitive dysfunction and neurodegenerative processes in their later years of life. Cognitive impairment was also linked to emotional and physical abuse, but more strongly linked to neglect, as lack of care and emotional support may have particularly harmful effects on cognitive development and resilience.

The relationship between trauma and cognition is complex and likely shaped by biological, psychological, and social factors, including genetic vulnerability, stress physiology, and social support. Nonetheless, the present findings underscore the importance of early identification of childhood trauma, particularly neglect, and the incorporation of trauma-informed approaches into dementia prevention and care pathways for women.

## Public significance statement

This study demonstrates that both emotional and physical neglect in childhood increases the risk of cognitive impairment later in life, particularly among women. The results suggest that childhood neglect is a key and modifiable risk factor for cognitive impairment and Alzheimer’s disease in women. Intervention in early life could thus help to reduce the rate of cognitive illness and reduce the likelihood of developing dementia later in life.

## Data Availability

The original contributions presented in this study are included in this article/supplementary material, further inquiries can be directed to the corresponding authors.
